# The State of the Art and Potentialities of UAV-Based 3D Measurement Solutions in the Monitoring and Fault Diagnosis of Quasi-Brittle Structures

**DOI:** 10.3390/s25165134

**Published:** 2025-08-19

**Authors:** Mohammad Hajjar, Emanuele Zappa, Gabriella Bolzon

**Affiliations:** 1 Department of Civil and Environmental Engineering, Politecnico di Milano, Piazza Leonardo da Vinci 32, 20133 Milan, Italy; 2 Department of Mechanical Engineering, Politecnico di Milano, Via Privata Giuseppe La Masa 1, 20156 Milan, Italy

**Keywords:** structural health monitoring, drones, digital image correlation, photogrammetry, time-of-flight, UAV, 3D measurement

## Abstract

The structural health monitoring (SHM) of existing infrastructure and heritage buildings is essential for their preservation and safety. This is a review paper which focuses on modern three-dimensional (3D) measurement techniques, particularly those that enable the assessment of the structural response to environmental actions and operational conditions. The emphasis is on the detection of fractures and the identification of the crack geometry. While traditional monitoring systems—such as pendula, callipers, and strain gauges—have been widely used in massive, quasi-brittle structures like dams and masonry buildings, advancements in non-contact and computer-vision-based methods are increasingly offering flexible and efficient alternatives. The integration of drone-mounted systems facilitates access to challenging inspection zones, enabling the acquisition of quantitative data from full-field surface measurements. Among the reviewed techniques, digital image correlation (DIC) stands out for its superior displacement accuracy, while photogrammetry and time-of-flight (ToF) technologies offer greater operational flexibility but require additional processing to extract displacement data. The collected information contributes to the calibration of digital twins, supporting predictive simulations and real-time anomaly detection. Emerging tools based on machine learning and digital technologies further enhance damage detection capabilities and inform retrofitting strategies. Overall, vision-based methods show strong potential for outdoor SHM applications, though practical constraints such as drone payload and calibration requirements must be carefully managed.

## 1. Introduction

The safety assessment and preservation of long existing structures, as part of infrastructure networks or historical heritage, relies on the continuous monitoring of their response to external actions [1,2,3,4,5,6]. The purpose of monitoring is to detect anomalies in the response and identify potential damage. The information collected from slender structures like bridges and bell towers is often related to ambient induced vibrations, e.g., due to traffic [4,7,8,9]. Displacements are mostly recovered in the case of massive structures like dams, which are subjected to seasonal changes in temperature and water levels [2,3]. In this context, particularly significant are relative displacements at joints that constitute the main non-linearity source of these facilities [3,10,11]. Relative displacements accompany the evolution of cracks in masonry buildings, a consequence of foundation settlements or non-destructive seismic events [12,13]. In structural assessments, the presence of fractures is a primary concern, as it typically indicates strength degradation. This is often accompanied by a reduction in the overall stiffness, which may lead to excessive deformations. For example, the alkali–aggregate reaction in concrete has been identified as a cause of strength deterioration in dams in [14,15]. In some cases, the source of unrecoverable displacements cannot be easily explained [16]. This highlights the importance of effective and continuous monitoring to obtain deeper insight into the damage state of the examined structure. This review focuses on UAV-based (unmanned aerial vehicle) 3D measurement solutions for structural monitoring. The main goal of these monitoring techniques is twofold: detecting cracks and quantifying their geometry, including crack opening and length, and estimating the relative displacements between adjacent elements of a structure. This manuscript reviews the most recent UAV-based measurement applications, where the emphasis is on the safety assessment of structures undergoing fracture and other degradation phenomena characterized by relative displacements. The search primarily considered recent papers (from the 2000s) on structural health monitoring, with a focus on concrete and fracture-related problems.

Traditionally, structural monitoring of massive quasi-brittle structures relies on instruments such as pendula, collimators, and callipers installed in dams [2] or strain gauges and Linear Variable Differential Transformers (LVDTs) placed in masonry walls [6,12]. In newly built or recently retrofitted facilities, a large network of sensors allows for the recovery of a large amount of data, providing a comprehensive overview of the health status and possible damages during construction. Otherwise, this information can be complemented by measurements performed by non-contact methodologies, such as mounting the relevant equipment on drones [17,18,19,20,21].

Computer-vision methods support the inspection of civil structures and infrastructures. Available techniques allow us to detect cracks, missing parts, and deteriorations and evaluate possible collapse mechanisms in zones of difficult access and in potentially critical situations [22,23,24]. Quantitative information at different accuracy levels, for different applications, can be recovered from full-field measurements depending on the equipment, measurement system, and surface preparation, as illustrated in this review paper. With technological developments, drones have gained widespread popularity as versatile tools for a variety of applications across research and industry. A key advantage of imaging techniques is that the optical sensor can be mounted on a UAV, enabling data collection from various positions.

The collected data can be used to calibrate simulation models of the structure, which typically employ numerical analyses performed mostly within a non-linear finite element context. The availability of displacement maps permits us to overcome the limitations due to uncertain boundary conditions and possible degradation of material properties over time, providing data suitable for implementing sub-structuring and model update strategies [25]. The digital twins of real constructions or part thereof can support the interpretation of the measurements and evidence any anomaly with respect to the expected trend [3,26].

In all cases, emerging procedures based on digital technology and machine learning tools allow us to improve the accuracy and reliability of predictive modelling and real-time monitoring, as well as to plan optimal retrofitting interventions in the event of progressively damaging phenomena [27,28,29,30,31,32].

Different vision-based techniques are reviewed, and their performance is compared in the context of structural assessments. The contribution of this review lies in identifying the strengths of each technique, summarizing the achievable accuracy of each method, and offering insights into potential future developments. In addition, critical factors that significantly affect the performance of each technique are highlighted.

## 2. Non-Contact Monitoring Techniques

With technological developments, non-contact measurements using vision-based techniques became popular, providing full-field monitoring of the region of interest. Examples of promising methods include 3D digital image correlation (3D-DIC), photogrammetry, and time-of-flight (ToF) sensors. DIC compares a sequence of images to determine the relative motion that have occurred. The setup for a DIC application consists of an image acquisition system, a speckled surface, and suitable lighting [33,34]. Photogrammetry uses a series of images acquired with a single camera from several locations to reconstruct a 3D model of the observed scene [35]. ToF sensors emit several infrared pulses and collect the return times along several directions in the field of view. The distance from the sensor to each point on the surface is then obtained based on the time needed for the pulse to be reflected back from the target [36]. A key advantage of vision-based techniques is that the optical sensor can be mounted on an UAV and can record information about the target from different locations. It should be noted that many optical sensors currently available on the market are capable of capturing frames at very high rates, often exceeding 100 frames per second. Therefore, the sampling rate should not present a challenge for monitoring civil engineering structures, which are often characterized by relatively low natural frequencies.

The ability of drones to move a sensor along the desired trajectory has the disadvantage that the data is not expressed in a single reference system. Furthermore, the rototranslations of drones can only be measured with non-negligible uncertainty. Some review articles discussed drone performance and payload. The authors in [37] mentioned that the UAV motion is sensitive to wind due to its low weight. Control and navigation algorithms can mitigate this issue under constant wind speeds, but fluctuations in wind speed and direction lead to a correction lag and thus affect image quality.

The usage of UAVs in civil engineering was reviewed in [38], dividing its applications into seven main categories: site safety management, city traffic management, disaster damage assessments, wireless communication, power distribution system monitoring, fluvial remote sensing, and structure health monitoring. Another review was performed in [39], which considered papers until 2019.

**Figure 1:**
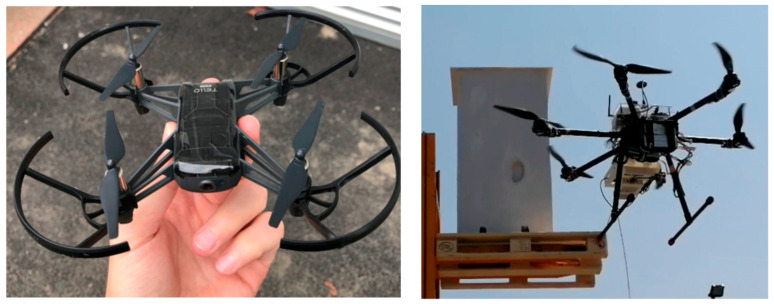
Example of UAVs equipped with optical sensors.

Various drone models could be employed for monitoring applications, ranging from the simplest and cheapest ones, which can be operated without any professional training or license, to the largest ones, which can be off-the-shelf or even custom devices. Of course, as the size of the UAV increases, so does the allowed payload, leading to the possibility of mounting not only simple digital cameras but also 3D scanners, infrared cameras, lidar sensors, and processing units. On the other hand, the use of larger drones imposes increasingly demanding operator training, as well as more complex flight permission procedures and costs. Sample drones that could be used for UAV measurements are shown in Figure 1; the left-hand side shows a small and cheap drone with an integrated camera, while the right-hand side depicts a larger and custom drone equipped with cameras and 3D scanning devices, and it is controlled by an on-board single-board PC. The review in [40] discusses the criteria for selecting a suitable drone, such as the remote range, the ability to fly without a GPS signal, the presence of source illumination on the drone and payload, and the capability of mounting an additional camera. While the most appropriate drones cost about USD 45,000, other drones with prices ranging from USD 2000 to USD 4000 were deemed acceptable. All these drones have a comparable flight time (20 to 30 min), support an additional flashlight to improve illumination, and have a remote-control range larger than 1000 m. According to the authors in [41], future developments are expected to enhance the reliability of vision-based applications under adverse weather conditions. It was reported in [42] that the main limitations for UAV uptake in civil engineering include flight endurance and payload, legislations and regulations (in some cases a special permission is needed for flying a UAV in public areas), lens distortion, weather conditions, and the volume of data to be processed.

The following applications highlight the importance of each of the aforementioned vision-based techniques in view of their combined use with UAVs.

### 2.1. Digital Image Correlation (DIC)

**Figure 2:**
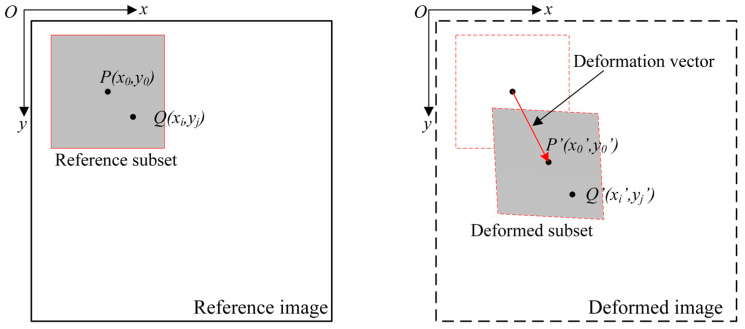
Illustration of subset tracking between the reference and deformed configurations [3].

The purpose of DIC is to find the displacement and strain fields of the target surface by processing the digital images acquired before and after the deformation occurs. The first image is frequently selected as the reference image that corresponds to the undeformed configuration. The basic concept works by tracking the location of image subsets between the reference and deformed images, as illustrated in Figure 2. The difference between the position of each reference subset and that of the corresponding deformed one yields one displacement vector.

**Figure 3:**
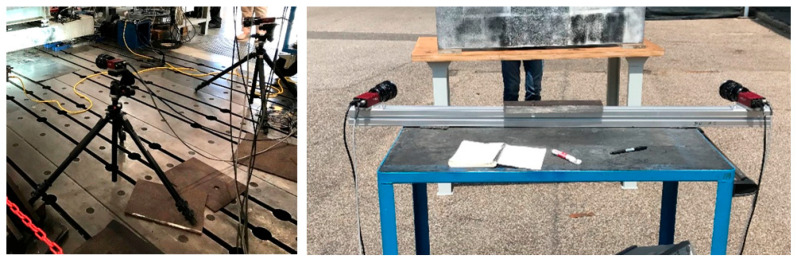
Cameras mounted on tripods (**left**) and fixed support (**right**).

The initial applications of DIC worked in two dimensions, computing pixel displacements along a planar surface [43]. Over the past two decades, research on DIC has grown substantially, driven by improvements in imaging technology and computational power [44]. DIC applications could be either two-dimensional (2D) or three-dimensional (3D). One camera is used in 2D DIC as the in-plane displacements are obtained. Three-dimensional DIC combines the concepts of 2D DIC with stereovision principles, requiring a minimum of two cameras to determine the 3D shape and deformations. DIC is highly effective in laboratory applications under controlled conditions, where cameras are mounted on fixed supports (Figure 3) and additional lighting can be used as needed [45,46,47,48,49,50,51,52,53]. Small crack opening displacements of 0.05 mm and 0.1 mm were measured in [47,53], respectively. These studies demonstrate the high performance of DIC in controlled laboratory conditions; however, when applied using a UAV in field, the accuracy is expected to decrease. This point is discussed in the next paragraphs.

**Figure 4:**
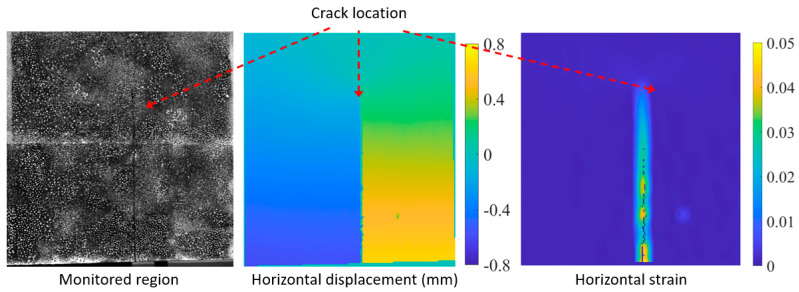
Sample results from an outdoor DIC application [54].

Shear tests were performed on prestressed concrete girders in the laboratory in [48], and it was mentioned that DIC provided more detailed kinematics of the crack compared to the used laser sensors and displacement transducers. The monitored region in Figure 4 corresponds to an extruded polystyrene panel loaded at its midsection. As a result, a crack develops and propagates. A key advantage of DIC is its ability to provide full-field deformation measurements, where both displacements and strains can be used to identify cracks, as illustrated in Figure 4. These displacement fields constitute an important step toward obtaining a digital twin. In [52], for instance, a reduced model of the monitored structure was proposed, with boundary conditions derived from the experimental data obtained using DIC. This approach was further enhanced in [54], where the crack opening displacement values were incorporated into an optimization procedure to determine the fracture parameters.

DIC features many applications for measuring displacements in large specimens. Target-tracking 3D DIC was proposed to examine the vibration of 21 m span bridges subjected to bidirectional loading in the laboratory [55], as well as the vibration of a pedestrian bridge [56]. The vibration responses measured with DIC were used to determine the modal parameters of the bridges. Three-dimensional DIC was also used in the laboratory to detect damage in steel beams using inverse analysis and topology optimization with a finite element model [57]. A 3D DIC system was developed to measure the vibrations of a lighting pole and a wind turbine [58]. When monitoring large structures, the camera’s field of view may not fully cover the region of interest. This limitation can be addressed through image stitching, as achieved by combining DIC with geodetic surveying in [59] and laser tracking in [60].

Cameras can be mounted on a UAV to capture images of the target area, providing a practical solution for large infrastructure that is often difficult to access. In [61], the behaviour of a repaired steel beam in a bridge was examined during and after the removal of the jacking support. In that application, the strains calculated by DIC were larger than those obtained with the strain gauges. The reason for this difference was attributed to an anomaly in the foil gauge measurement. In another study, two cameras mounted on a UAV were used to measure displacements in two bridges over a period of 10 months [19]. A discrepancy of 15% was found between the measurements obtained with DIC and a dial calliper, referring to an absolute error of 0.34 mm. In [62], two cameras mounted on a UAV were used to obtain strains of a rotating wind turbine. In [63], the dust on the bridge beam acted as a speckle pattern. A series of studies was conducted on the use of DIC with UAVs in recent years [64,65,66]. The authors first implemented a vision-based position estimation framework to keep the UAV steady in the desired position [64]. The proposed approach utilizes fiducial markers attached to the surface in order to precisely locate the target area. This system was later validated in a laboratory study [65], as it was used to measure deformations of a prestressed railroad tie loaded in four-point bending. In [66], the system was employed to monitor steel girders in a bridge. The results were not accurate as the obtained strain was larger than the maximum expected value, and it was not possible to distinguish between the loaded and unloaded bridge conditions. This inaccuracy was due to the noise mainly caused by camera vibrations, despite the fact that isolation pads were used. The studies cited above demonstrate that DIC can achieve good accuracy even with cameras mounted on a UAV, even if it is not possible to reach the same accuracy granted by the same DIC system mounted on a tripod, as shown in Table 1.

Some papers presented a literature review about the usage of UAV-aided vision-based measurement techniques on civil engineering structures [67,68,69,70,71]. DIC-based inspection of suspension, masonry, concrete, and steel bridges is considered in [67]. The cost and time saved using vision-based techniques compared to traditional monitoring techniques are analyzed in [69].

### 2.2. Photogrammetry

In photogrammetry, a series of photos is acquired by one camera from different positions and orientations with respect to the monitored structure. The image processing required to recover the 3D shape of the target involves the following:

1. The identification of a set of features present in each image, typically performed with the scale-invariant feature transform (SIFT) algorithm [72].
2. Robust features identification and tracking, normally obtained with the random sample consensus (RANSAC) algorithm [73], to iteratively compute the rigid motion (rotation and translation) of one camera with respect to another, taking corresponding keypoints in the images as a starting point.
3. Camera alignment and automatic calibration, obtained by finding correspondences between the features in several images [74].
4. Three-dimensional reconstruction, obtained by means of stereo triangulation between pairs of images sharing a set of features [75].

**Figure 5:**
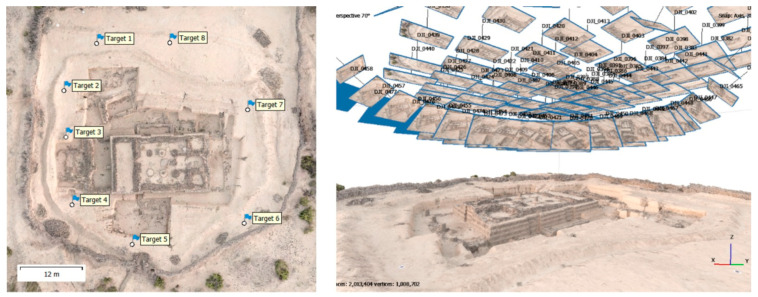
Sample model obtained with photogrammetry: Top view (**left**) and side view (**right**).

Due to the robustness in the process of feature extraction and matching, no issues are faced when the distance from the camera to the structure slightly varies during acquisition. Photogrammetry, also referred to as structure from motion (SfM) reconstructs scene geometry, camera positions, and orientations without requiring a predefined 3D point network [75], making it particularly advantageous for UAV-based image acquisition [76]. Figure 5 presents a 3D model obtained with photogrammetry, depicting an ancient building in an archeological park. On the left side (top view), the target points that are used for referencing and scaling can be seen. On the right side, images are displayed in 3D at their estimated camera positions, illustrating the multi-view approach in photogrammetry.

Some studies have utilized UAV photogrammetry, primarily focusing on 3D model generation. UAV photogrammetry was employed in various fields, including studies on historical buildings [77,78,79], archeological sites [80,81], and façade structures [82]. In [83], a 3D model of a building was constructed by applying photogrammetric techniques, achieving an accuracy of up to 80 cm. This large error is due to the low resolution of the camera used (1.15 MP) and the large size of the surveyed structure (64 m by 58 m). UAV photogrammetry was used to create 3D models of specific segments in two bridges [84,85]. Additionally, image processing algorithms were implemented to detect cracks in the images and locate them in the 3D models. Under a working distance of 1 m in [84], crack openings varying between 2 mm and 4 mm were identified with a mean error of 13%, while openings less than 1.5 mm could not be accurately detected. The errors dramatically increased when the working distance was doubled. In [85], the generated point cloud had an average accuracy of 0.7 mm, and crack openings extending from 3 mm to 20 mm were identified with a mean error of 1 mm.

In [75], the results obtained with UAV photogrammetry were compared to those obtained with laser scans acquired from fixed station points. Reference points on the surface, known as ground control points (GCPs), were used for orienting and scaling the model. The error between terrestrial laser scanning and photogrammetry was 30 mm. UAV photogrammetry was used to build a 3D model of the Ridracoli dam in Italy [86]. An accuracy of 15 mm was found with respect to measurements by laser scanners. The vertical contraction joints could not be observed by analyzing the horizontal sections of the 3D scene. However, they could be identified in the red–green–blue (RGB) information of the point cloud, being darker than the surrounding region. The accuracy of the main photogrammetry applications is summarized in Table 2. This table highlights the importance of additional processing steps in enhancing the accuracy of photogrammetric measurements. Another study on the Ridracoli dam was performed [87], with the purpose this time on finding the optimal number and location of GCPs which provide the best 3D scene reconstruction. Square markers with a width of 40 cm were attached to the surface and used as GCPs. A mean vertical error of 10 cm (which is less than 1% of the structure height) was considered acceptable. The results showed that the error is sensitive to the number of GCPs along the vertical direction, so placing the GCPs at different elevations increases the accuracy. A qualitative assessment was performed based on a photographic survey of a portion of a dam in Portugal [88]. Image classification was used to detect anomalies, such as recognizing calcium carbonate deposits caused by water leakage below horizontal cracks.

### 2.3. Tof and Laser Scanners

**Figure 6:**
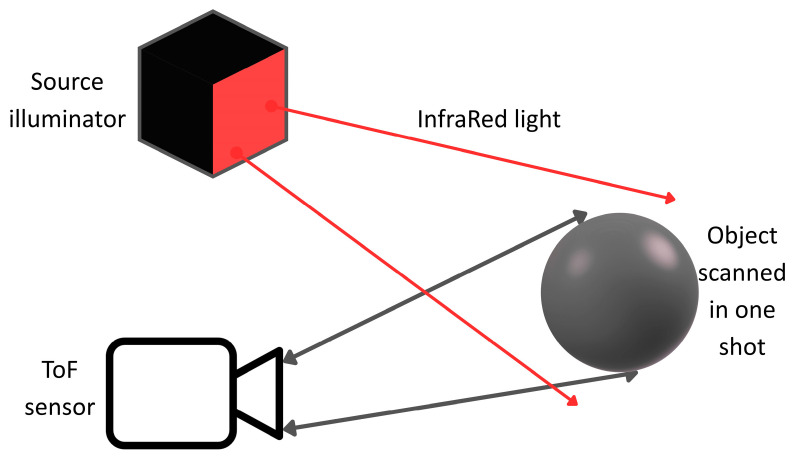
Typical setup of a ToF device.

**Figure 7:**
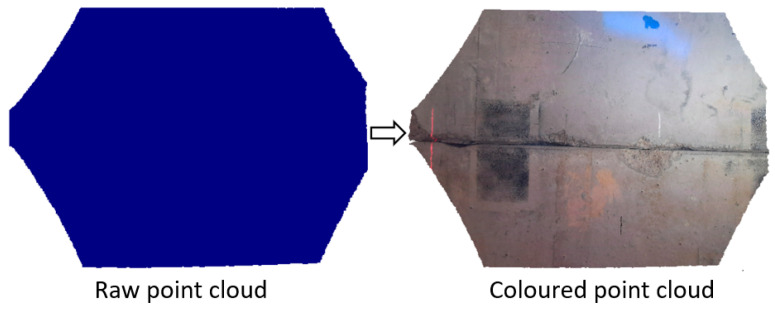
Raw point cloud (**left**) and the corresponding coloured cloud (**right**).

Time-of-flight (ToF) devices include an infrared (IR) light emitter that illuminates the scene and an imaging sensor that detects the reflected light [89]. The typical setup of a ToF device is illustrated in Figure 6, showing a sketch of the light source illuminating the scene along with the sensor scanning the object in one shot. The integrated depth camera contains a ToF chip, in which the pixels capture the phase shift between the emitted signal and the reflected IR signal [90], which is linked to the time taken by the emitted radiation to reach the target and be reflected back. The distance from the IR emitter and the object is then calculated based on the recorded phase shift [91]. A 2D depth map is produced, where x and y represent the coordinates of each point in pixels, and the depth for each pixel is stored. The measured depth value corresponds to the Z coordinate (in mm) of the sampled point, and it differs from the Euclidean distance [92]. The X and Y coordinates (in mm) of each point can be determined using the camera’s intrinsic parameters, enabling the generation of the 3D point cloud. Some ToF devices feature both a colour camera and a depth camera. By utilizing the intrinsic and extrinsic camera parameters, it is possible to generate a coloured point cloud, in which the colour information is added to the 3D cloud. The advantage of using a coloured point cloud is displayed in Figure 7, where the surface texture is clearly visible and can be used for displacement calculation.

The study in [93] thoroughly evaluated the performance of four ToF devices. The parameters studied included the measurement error, the standard deviation, and the edge noise. Table 3 reports the depth error as a function of the increasing measurement range. The values represent the average error obtained with the four ToF devices. As also observed in [93], the error increases with the operating range.

ToF sensors have been used in a wide range of applications other than civil engineering, including robotics, and clinical purposes such as human motion detection and posture recognition [94,95,96,97,98]. This methodology was also used as a part of a low-cost crack monitoring system [99]. ToF cameras can be mounted on drones to perform measurements of the 3D shape of portions of the target structure. Accurate results were obtained by employing techniques apt at mitigating the sensor vibration and displacement caused by the UAV motion [100]. These approaches allow for the identification of the geometry of defects in concrete, such as spalling and swelling, with an accuracy of less than 4 mm in comparison to a high-resolution 3D scanner [101]. The use of UAVs for the inspection of bridges and tunnels was reviewed in [102], noting that IR-based inspections remain underexplored in this field.

The purpose of this table is to illustrate the variations in maximum achievable accuracy across the different techniques. The second column of this table shows the smallest detected crack or defect reported among all references for each technique. For the corresponding reference, the average crack size is then calculated and reported. For example, the smallest measured crack with UAV-based DIC applications is found in reference [19]; the value reported in the second column represents the average size of the crack measured in that reference. As seen in Table 4, it is evident that DIC possesses the finest measurement accuracy compared to photogrammetry and ToF. Additionally, it is important to highlight that further processing steps are essential in improving the accuracy of the displacements derived with photogrammetry and ToF. Table 4 presents the smallest detectable crack or defect size for each technique.

Laser scanning technology is similar to ToF in terms of basic working principle. Nonetheless, ToF sensors emit a laser pulse that can survey the entire scene at once, while traditional laser scanners emit a signal beam to explore the surface point by point with a scanning mechanism. Due to this reason, laser scanning is not always suitable for UAV-based measurements. However, laser scanning allows for measurements over large distances (hundreds of metres), while ToF sensors have measuring ranges limited to a few metres. Scans from multiple locations are integrated together in laser scanning, and the point clouds from each local coordinate system are registered into a global coordinate system. The errors resulting from the registration process could reach 14 mm [103]. In addition, laser scanning could be integrated with photogrammetry [104,105]. A study on two bridges was conducted in [106], and a discrepancy ranging from 7 mm to 30 mm was found between the point clouds produced by photogrammetry and laser scanners. Laser scanning was applied to monitor the surface of a concrete beam, which was tested in the laboratory [107]. Loading was applied in steps, and a scan of the surface was completed for each step. In another experimental study [108], a fixed laser scanner was used to monitor the deformations of an arch structure. The error in the point cloud data was 0.8 mm. Laser scanning is used in the construction industry as well [109]. The usage of traditional instruments (including Linear Variable Differential Transformers (LVDTs), laser sensors, accelerometers, and strain gauges) and non-contact techniques (such as DIC and laser scanning) during load tests of bridges is recalled in [110]. It was noted that non-contact methods are not commonly used in this context.

LiDAR (Light Detection and Ranging) systems emit a large number of laser pulses toward the target area [111]. UAV-based LiDAR systems can merge multiple scans acquired from different positions to generate a 3D point cloud. Merging these scans requires estimating the UAV’s trajectory with six degrees of freedom. Trajectory estimation is usually named “odometry”, and it is a fundamental chain in UAV-based LiDAR systems. The fidelity of the odometry affects the accuracy of the obtained 3D point cloud. Different approaches could be performed for odometry; a LiDAR-only odometry determines the UAV’s position by analyzing consecutive LiDAR scans [112]. Alternative methods either integrate an IMU (inertial measurement unit) with LiDAR or employ multiple LiDAR systems. However, using multiple LiDAR systems or additional sensors increases computational demands.

LiDAR measurements are not reliable in environments with sparse geometric features or repetitive topographical characteristics, such as long tunnels [112]. Similar challenges may also arise in dams. In some cases, fusion of LiDAR with RGB cameras might be the solution [112], as the RGB cameras provide colour and texture information. This is similar to the process used with ToF devices, where depth and colour frames are merged to create a coloured point cloud. However, the latter is a much simpler procedure and requires less computational effort. Another limitation of LiDAR systems is the large amount of data that needs to be processed [112].

It was mentioned in [113] that the use of UAV-based LiDAR systems is occasional, as such devices are not considered suitable for high-precision monitoring applications. This is in the agreement with the findings in [114], where the performance of some of the available low-cost LiDAR scanners was evaluated. In this study, experiments were conducted to measure the distance between the fixed scanner and a flat target surface. In the outdoor experiment, the error in the distance measurements ranged from 1 cm to 3.7 cm. In [115], 3D models of a bridge were created using two distinct methods: UAV photogrammetry and UAV-based LiDAR. The error in the 3D point cloud generated with photogrammetric techniques was around 2.5 cm, while that obtained with the LiDAR ranged from 5 to 7 cm. Moreover, a solid-state (fixed) LiDAR system was used in [116] to survey the geometry of a ballast system in the laboratory. Each scan was acquired with the LiDAR system mounted on a trolley attached to the track. The error in the measurements ranged from 5 to 7 mm. According to the previously cited studies and the errors found with the LiDAR measurements, it could be said that such systems are not the best choice for high-precision monitoring applications in structural engineering. For this reason, UAV-based LiDAR applications are not thoroughly surveyed in this manuscript.

## 3. Lighting Issues

Many issues are present in the application of imaging techniques, such as the image noise that is associated with the sensor’s resolution, illumination variability, computational load, and the integration and calibration of multiple sensors. In field applications, vision-based techniques might face challenges under specific environmental conditions, with sunlight disruptions being a major factor. This section reviews the main issues caused by illumination variation, their impact on measurement accuracy, and the possible mitigation techniques. For instance, DIC relies on grey intensity conservation, assuming that a pixel’s grey value distribution remains consistent between the reference and deformed images. However, in field applications, variations in lighting could occur due to moving clouds, camera shadow, changing weather conditions, a large field of view, etc., thus affecting the accuracy of DIC measurements. This issue was reported in [56] during the monitoring of displacements in a pedestrian bridge, where the camera exposure time had to be adjusted during image acquisition due to moving clouds. A similar problem was encountered during bridge monitoring in [19], resulting in overexposed or blurry images, which increased the absolute error from 0.05 mm to 0.34 mm.

The impact of illumination variations on DIC measurements was investigated in some studies. In [117], images were digitally modified to introduce changes in lighting conditions. The deformation scenarios consisted of undeformed samples and deformed samples with homogeneous and non-homogeneous strains. Acceptable measurements were achieved when the brightness in the target image was uniformly modified. However, large errors were detected when lighting variations were introduced across different areas of the image. In a similar study [118], images corresponding to rigid body translation were considered. The image’s brightness was adjusted by a constant offset, and the measurements were acceptable for a mean grey level between 32 and 250. However, a sharp increase in the errors was observed at lower grey levels. These findings align with the formulation-based statements in [34].

Some studies were performed to address the issue of light variation in DIC applications. A method based on the Fourier transform was used to filter illumination noise, and its accuracy was validated both indoors and outdoors by monitoring an aircraft panel subjected to an impact load [119]. Brightness correction was used in monitoring cracks in a cube made of refractory castable, with global DIC being used instead [120]. Grey-level disturbances are caused by the experimental conditions (temperature and humidity) and by the development of cracks. The brightness value at every pixel in the reference image was adjusted by means of brightness and contrast correction functions. Such techniques are useful when illumination changes between images, but they do not solve the issue of nonuniform illumination within the same image. It should be noted that the most advanced DIC tools can accommodate the drift in lighting conditions [33], provided that no abrupt brightness distribution arise from one image to another.

**Figure 8:**
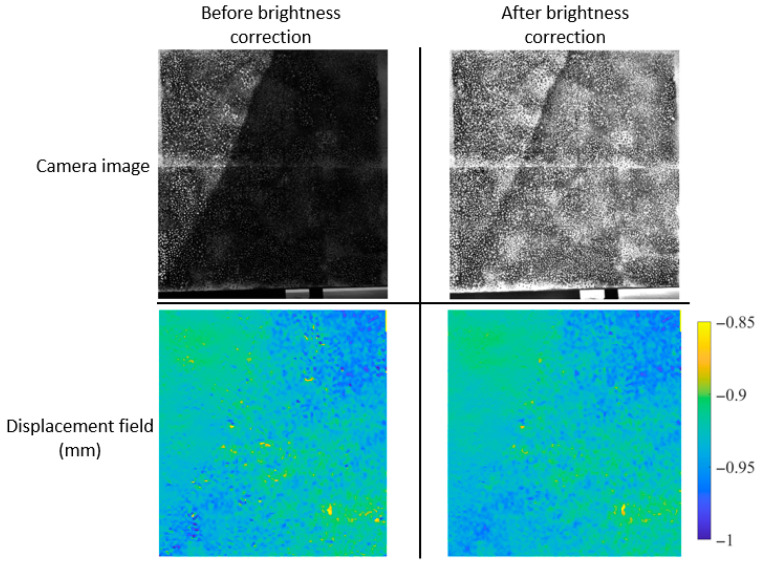
DIC results for a sample image containing a shadow and its corresponding compensated one [54].

In [121], correction for irregular intensity was achieved by subtracting the background from the original image. The study involved laboratory tests on an aluminum plate under uneven illumination, including rigid body rotation and three-point bending. The correction method reduced the measurement error by an order of magnitude. Another brightness correction method was developed in [122], based on the assumption that the image is composed of an illumination component and a reflection component. The needed information, such as the texture or speckle pattern, is represented by the reflection component. This study did not include a DIC application; however, the developed method was tested on images of various landscapes. Another correction method based on Retinex theory [123] was applied in [124], where first a numerical simulation was performed on digitally modified images to represent the nonuniform intensity distribution. The mean error and the standard deviation were calculated, and a reduction in these quantities was observed after correcting for nonuniform illumination. Laboratory experiments were then conducted including rigid motion and uniaxial testing. A cold lighting source was placed in front of the lighting device to vary the brightness levels. The results of the study in [124] showed that the brightness correction approach reduced the errors and provided smoother displacement fields. In another study [54], outdoor experiments were conducted to simulate the presence of shadows in the monitored region. Image brightness adjustment was then applied, reducing the disturbances in the measured displacements. Figure 8 presents a sample image from this study [54] along with the corresponding vertical displacement fields obtained using DIC. In this test, the monitored region was fixed, and a uniform displacement field was therefore expected. The objective was to assess the noise level in the DIC measurements, both before and after applying image brightness correction. It can be seen that the displacement field on the bottom right side of Figure 8 is smoother than that of the bottom left side. This shows the improvement obtained using the brightness correction algorithms. As a quantitative assessment of the brightness enhancement technique, the standard deviation of the displacement field was calculated in [54], and the values are shown in Table 5. A reduction in the standard deviation can be seen after applying the brightness modification approach, indicating a smoother displacement field.

Similar issues could occur with ToF measurements, as the sunlight IR radiations may create disturbances to the signal detected by the sensor. This could be mitigated by using ToF devices that operate at a wavelength of 940 nm [101], where sunlight is substantially diminished by the filtering effect of the atmosphere [125]. Other sensors used in [101] had wavelengths smaller than 900 nm, and their accuracy remained unchanged when exposed to daylight without direct sunlight. Tests were performed in [94] under direct sunlight, and acceptable results were produced for a measuring range not exceeding 1.5 m.

## 4. Learning Tools

The information gathered by vision-based sensors can be improved by the use of machine learning and deep learning algorithms, allowing damage detection and quantification. Post-processing techniques can also overcome limitations connected with on-site acquisition of images under variable environmental conditions. Such tools were used to automatically identify cracks or defects in the images of a concrete surface [126,127,128]. Pictures of a laboratory frame and of an elevator tower were acquired by a camera mounted on a UAV [129]. Features identified with a deep learning neural network were used to compensate for camera movement and to calculate the relative displacements. Similarly in [130], features were detected in each recorded frame in order to track displacements between consecutive frames. A bridge inspection was carried out in [131] and defects such as efflorescence, water leakage, spalling, and discoloration were identified. A machine learning framework was applied to images acquired with a UAV in order to measure the crack lengths in a bridge [132]. In [133], an image processing algorithm was implemented to detect cracks in a concrete bridge from images collected by a UAV. In a similar study [134], deep learning was applied to detect and quantify crack openings. Summary data of these two studies is reported in Table 6. In [135], deep learning was applied to detect cracks in the images captured with a UAV. The deep learning model was previously trained on a dataset of images containing dam cracks. Moreover, deep learning was used in [136] to obtain vibration information in tall buildings. In [137], reference targets with specific patterns were installed on the tested structure. These targets were necessary, as they served as identifiable features whose displacements were tracked. Errors of several millimetres (from around 4 to 10 mm) were detected at the reference target locations.

Several review articles on the application of learning techniques for monitoring civil engineering structures have been published recently [138,139,140,141]. It was also mentioned in [141] that only a few studies focused on obtaining the crack geometry (its length and opening). This is mainly due to the fact that, to obtain metric information about the crack, it is necessary to estimate the instantaneous position and orientation of the camera with respect to the target surface, which are hardly available in UAV-based measurements. An additional advantage of learning tools is that they can be integrated with other image processing techniques. This combined approach has been shown to improve accuracy, as previously noted in Table 2.

## 5. Conclusions

In general, the conducted literature review confirms that vision-based techniques are suitable for outdoor structural monitoring and fault diagnosis. However, factors such as drone size and payload capacity should be considered. A large UAV is required for 3D DIC applications to mount the rigid beam, which supports the two cameras, ensuring that their relative position and orientation remain fixed, to guarantee the validity of the calibration. In addition, the drone should be equipped with a power supply and a computer to control the image acquisition process. A medium-sized drone with a computer on board can be used also for ToF recordings. The photogrammetry technique provides the greatest flexibility in terms of the type of drone that can be used, since only one camera is needed, and it could be remotely controlled. Thus, a drone costing around USD 1000 may suffice for photogrammetry applications, whereas ToF and 3D-DIC techniques typically require a more advanced drone, priced at approximately USD 4000–USD 5000 or higher. However, drone technology is rapidly evolving, and according to information from UAV manufacturers, more efficient platforms are expected to become available in the near future, enabling the integration of ToF devices on lower-cost drones (approximately USD 2000). One point to be highlighted concerning photogrammetry applications is that, if the UAV is not equipped with a GPS device to tag each acquired image with the GPS coordinates, the distance between at least two reference points on the surface must be available in order to adequately scale the model.

In terms of accuracy, DIC performs best, achieving submillimetre precision, whereas photogrammetry and ToF typically reach accuracies within a few millimetres. In particular, DIC measured crack openings as small as 0.05 mm using a 2 MP camera, whereas photogrammetry, even with a 15.6 MP camera, captured a minimum crack size of approximately 2 mm (Table 4). Learning-based tools are also of great importance, with promising results reported when integrated with other techniques, such as photogrammetry. Different techniques with various hardware are compared in this review. The experimental setup (configuration, sensor placement, data acquisition, and processing procedures) has an influence on the final results; however, this information is not always available, and the comparison values given in the tables are therefore meant to be indicative. A main difference between DIC and the other two methods (ToF and photogrammetry) is that the displacements are originally provided in the DIC output, while the other techniques only produce a 3D reconstruction of the surface. Thus, an additional processing step, with associated uncertainty, is required to obtain the displacements. This could be performed either by point cloud registration or by identification of features on the surface. The main issues with ToF devices are depth measurement uncertainty (around 3 mm for sensors suitable for UAV-based structural monitoring) and a low camera resolution, as the most common ones do not exceed 1 Megapixel. New generations of ToF devices are expected in the near future, with improvements that will greatly impact the measurement accuracy. UAV-based LiDAR systems were also considered in this review; relatively large errors ranging from 1 cm to 7 cm were found [114,115]. In addition, these systems present a challenge due to their increased computational cost.

To provide a broad view on the current challenges and future developments, the following comments are provided to improve the new research in this field. Regarding light variations, for example, currently compensation techniques are already present and provide an acceptable result. In addition, mitigation techniques for the UAV motion and drifting are present and could be used. However, significant improvements are needed in the following areas: integrating multiple sensors and improving the UAV position and trajectory estimation (by exploiting global positioning accuracy, as well as magnetometer and IMU data). These developments would require a higher computational effort, and further advancements in data processing are therefore anticipated. Moreover, it should be noted that the use of multiple sensors necessitates careful calibration and synchronization.

All the techniques mentioned in this review retrieve full-field information useful for calibrating continuously updated numerical models (digital twins) of the investigated systems and capturing the evolution of their structural response over time. The reliability of these predictions depends on the accuracy of the measurements, as well as on uncertainties about the actual geometry, element connections, and out-of-sight details. Therefore, no acquisition method outperforms the others in general, but the optimal choice comes from a balance of different and sometimes conflicting requirements.

## Figures and Tables

**Table 1:** Precision of DIC measurements: cameras on tripods vs. a UAV [19].

	Average Noise (mm)	Standard Deviation (mm)
Cameras on tripods	0.01	0.02
Cameras on a UAV	0.05	0.05

**Table 2:** Accuracy of photogrammetry measurements in different applications.

Reference Number	Application Details	Quantity Measured
[75]	UAV photogrammetry	3D model: accuracy of 30 mm
[83]	UAV photogrammetry	3D model: accuracy up to 800 mm
[84]	UAV photogrammetry + image processing + back-projection	Measured crack openings: 2 to 4 mm (mean error 13%)
[85]	UAV photogrammetry + image processing + back-projection	Measured crack openings: 3 to 20 mm (mean error 1 mm)
[86]	UAV photogrammetry	3D model: accuracy of 15 mm

**Table 3:** Average depth error in the ToF measurements in [93].

Measurement Range	Depth Error
1 m	1 mm
1.5 m	2.4 mm
2.5 m	3.4 mm
3 m	3.7 mm

**Table 4:** Comparison of the accuracy of the different vision-based techniques in UAV-assisted measurements.

Technique	Smallest Measured Crack/Defect (mm)	Camera Resolution	Reference Number
DIC	0.05	2 MP	19
Photogrammetry	2	15.6 MP	84
ToF	3.5	0.37 MP	101

**Table 5:** Standard deviation of the DIC displacements obtained before and after image brightness correction [54].

	Before Correction	After Correction
Horizontal displacement (mm)	0.0145	0.0128
Vertical displacement (mm)	0.0164	0.0121

**Table 6:** Summary data of the inspections in [133,134].

Reference Number	Method	Measured Crack Opening	Error
[133]	Multiple image processing steps	8 mm	5%
[134]	Deep learning tools	2.7 mm	Not reported
